# On the Action of Hydrate of Chloral

**Published:** 1871-08

**Authors:** L. A. Harcourt

**Affiliations:** Chicago


### Correspondence.

CHICAGO, July 29th, 1871.

PROF. J. F. MINER,

DEAR SIR :—In the June number of your journal is a report of a meeting of the Albany County Medical Society, the following paragraph occurs :—"Dr. C. D. Mosher wished to know the experience of the members with regard to the hydrate of chloral, he having lost a patient by the use of one grain."

Now this is, to say the least, a very indefinite statement. Nothing is said of the age, sex or condition of the patient; but simply that he, she or it, died from the use of one grain of the chloral hydrate. We are left to infer that one grain of what is fast becoming a popular drug, may prove fatal at any time, and in any case. This is at variance with my experience in the use of this remedy. I will give one or two cases in point.

Case I.—Mrs. T., aged 22 years, married, the mother of two children, the youngest six months old, on the night of March 15th, 1871, immediately after coition with her husband, injected into the vagina a strong solution of alum, and was instantly seized with excrutiating pain in the region of the womb, that organ apparently being thrown into violent spasms. The solution was quite cold. I saw her about fifteen minutes after the operation. She was in the most terrible agony I ever witnessed, fairly writhing with pain. She suffered more in five minutes than any woman could in the worst imaginable case of labor. Her face was flushed and contorted, her eyes glaring, teeth chattering, pulse high and bounding, and the mind in a state of phrensy, bordering upon delirium. I instantly administered thirty grains of the hydrate of chloral, injected warm water into the vagina, and applied flannel cloths, rinsed out of hot water, and saturated with a mixture of turpentine, olive oil and laudanum, over the hypogastric region. In fifteen minutes repeated the dose of chloral, determined to allay the pain if possible. This afforded marked relief for a time; but the paroxysm returning, I exhibited the chloral in fifteen grain doses every half hour or hour as required, until she fell asleep. She took, in all, ʒijss of chloral, and slept from twelve or one o'clock till morning. I should have mentioned that the accident—if such it may be called—happened about nine o'clock at night. The next day there was evidence of acute metritis, which yielded to the usual remedies. In a week I dismissed the case, and in another she was herself again. I watched the case with peculiar interest, having read a day or two before of a death from the same cause.

Case II.—Thursday March 30th, 1871, was called to see Mrs.

Lavin, Peoria street, aged 27 years, married, the mother of three children, the youngest two days old. The day after her confinement she took a cathartic which operated harshly. Wednesday night she got out of bed alone to go to stool, fell on the floor, and remained in an insensible state for more than half an hour. She had only her night dress on, and when found was chilled with cold and quite sick. Next day she was taken with convulsions, in one of which I found her. Chloral in half drachm doses controlled the convulsions and prevented its return. In this case, also, as might be expected, there was metritis, which yielded to appropriate treatment. The woman made a good recovery. She had been attended by a german midwife, from whom I could learn little of her labor.

I could mention many cases in which I have used chloral in doses of from five to thirty grains, in all of which the result was pleasant and satisfactory. Have heard Prof. Miller of Rush Medical College, Chicago, speak highly of its use in mid-wifery, and relate cases in which he believed convulsions had been forestalled by its timely administration.

I would like to know something of the history of Dr. Mosher's case, being skeptical as to the fatality of chloral in doses of one grain.

Truly Yours,

L. A. HARCOURT.

